# Data from a photovoltaic system using fuzzy logic and the P&O algorithm under sudden changes in solar irradiance and operating temperature

**DOI:** 10.1016/j.dib.2018.11.023

**Published:** 2018-11-09

**Authors:** Carlos Robles Algarín, Omar Rodríguez Álvarez, Adalberto Ospino Castro

**Affiliations:** a Universidad del Magdalena, Facultad de Ingeniería, Carrera 32 No 22 – 08, Santa Marta, Colombia; b Universidad de la Costa, Facultad de Ingeniería, Calle 58 No 55 – 66, Barranquilla, Colombia

## Abstract

This article presents the data of a PV system using fuzzy logic and the perturb and observe (P&O) algorithm, under sudden changes in the solar irradiance and the operating temperature of a PV module. The mathematical modeling of the PV module, the dc-dc converter and the fuzzy and P&O controllers were discussed in our previous work entitled "Fuzzy logic based MPPT controller for a PV System" (Algarín et al., 2017) [1]. Data are presented for six cases with different operating conditions: in the first case the two controllers were evaluated for standard test conditions with constant solar irradiance of 1000 W/m^2^ and constant temperature of 25 °C. In the remaining five cases sudden increases and decreases were made in the operating conditions of the PV module. Finally, the theoretical data of the PV system are presented, which can be used as a reference to analyze the data obtained with the two controllers in the six cases. Data are provided in Supplementary material in Tables 1–7.

**Specifications table**

**Table:** 

Subject area	*Energy*
More specific subject area	*Photovoltaic Solar Energy*
Type of data	*Table, Excel files*
How data were acquired	*Numerical simulations based on MATLAB/Simulink software for a PV system consisting of a 65 W PV module, buck converter, 12 V battery and two Maximum Power Point Tracking (MPPT) algorithms (fuzzy logic and Perturb and Observe).*
Data format	*Filtered and analyzed*
Experimental factors	*The data obtained for the variables of the PV system were resampled in 1/1000 times in order to obtain shorter data vectors. Subsequently, the data vectors of the power, current and voltage of the PV system were exported to an Excel file.*
Experimental features	*The data of the PV system were obtained for sudden changes in the solar irradiance and the operating temperature of the PV module. The simulation data obtained with the standard P&O controller and the fuzzy controller are presented.*
Data source location	*Universidad del Magdalena, Santa Marta, Colombia*
Data accessibility	*Data are provided in supplementary materials with this article*
Related research article	*C.R. Algarín, J.T. Giraldo, O.R. Álvarez, Fuzzy logic based MPPT controller for a PV System, Energies 10 (12) (2017) 2036.*https://doi.org/10.3390/en10122036

**Value of the data**

• With these data, readers can make comparisons with other algorithms used to track the maximum power point in a PV system. This saves time for comparisons in different weather conditions [2], [3].
• These data are useful for the implementation and experimental evaluation of MPPT controllers with high efficiency [4], [5], [6].
• The data obtained can be used to train MPPT controllers using different intelligent control techniques such as neural networks.
• These data are useful to analyze in detail the behavior of a PV system under sudden changes in the solar irradiance and the operating temperature of the PV module.

## 1 Data

This paper presents the numerical data obtained from the modeling and simulation of a PV system consisting of a 65 W PV module, a buck converter, a 12 V battery, a standard P&O controller and a fuzzy controller; under sudden changes in the solar irradiance and the operating temperature of a PV module. The simulations were performed on a computer with Windows 10 pro operating system, Intel Core i5-6500 processor and 8 GB RAM. Table 1 shows the specifications of the PV system used in the simulations.

**Table 1:** Specifications of the PV system.

Parameter	Value
PV module type YL65P-17b	65 W
Voltage at the maximum power point (V_MPP_)	17.5 V
Current at the maximum power point (I_MPP_)	3.71 A
Short-circuit current (I_sc_)	4 A
Open circuit voltage (V_oc_)	21.7 V
Temperature coefficient of voltage (T_cv_)	−0.0802 V/°C
Temperature coefficient of current (T_ci_)	0.0024 A/°C
Buck converter	
Inductor	410 μH
Capacitor	280 μF
Switching frequency	20 kHz
Fuzzy controller	
Inputs	2 (Error and change of error)
Output	1 (Increment in duty cycle)
Membership functions	5 (Triangular)
Fuzzy rules	25 (If-then)
P&O controller	
Inputs	2 (Voltage, current)
Output	1 (Duty cycle)

## 2 Experimental design, materials, and methods

In order to obtain the voltage, current and output power of the PV system for sudden changes in operating conditions, dynamic profiles were used for the solar irradiance and the operating temperature of the PV module. Using a fuzzy controller and the P&O algorithm, six (6) test cases were defined:

Case 1: Controllers were evaluated for standard test conditions, with a constant solar irradiance of 1000 W/m^2^ and constant operating temperature of 25 °C. Table 1 of Appendix A shows the data obtained for an approximate simulation time of 1 s with 1197 samples.
Case 2: Controllers were evaluated for a constant operating temperature of 25 °C and a solar irradiance signal that increases in different instants of time. This signal is between 200 W/m^2^ and 1000 W/m^2^ with increments of 200 W/m^2^. Table 2 of Appendix A shows the data obtained for an approximate simulation time of 1 s with 1200 samples.
Case 3: In this case the controllers were evaluated for a constant operating temperature of 25 °C and a solar irradiance signal between 1000 W/m^2^ and 200 W/m^2^ with decreases of 200 W/m^2^. Table 3 of Appendix A shows the data obtained for an approximate simulation time of 1 s with 1201 samples.
Case 4: In this case the controllers were evaluated for a constant solar irradiance of 1000 W/m^2^ and an operating temperature that increases in different instants of time with increments of 25 °C. This signal is between 0 °C and 100 °C. Table 4 of Appendix A shows the data obtained for an approximate simulation time of 1 s with 1188 samples.
Case 5: Controllers were evaluated for a constant solar irradiance of 1000 W/m^2^ and an operating temperature that is decremented from 100 °C to 0 °C with decreases of 25 °C. Table 5 of Appendix A shows the data obtained for an approximate simulation time of 1 s with 1200 samples.
Case 6: Controllers were evaluated for changes in operating temperature and solar irradiance. For the two signals, dynamic profiles with sudden increases and decreases at different instants of times were used. Table 6 of Appendix A shows the data obtained for an approximate simulation time of 1 s with 1191 samples.

Finally, Table 7 of Appendix A shows the theoretical data of the PV system; taking into account the values that were used for the solar irradiance and the operating temperature in the six cases described. These values can be used as a reference for the reader, in order to analyze the data presented for the two controllers.
